# Supplementary material for: NSm is a critical determinant for bunyavirus transmission between vertebrate and mosquito hosts
Source: Nat Commun. 2025 Jan 31;16:1214. doi: 10.1038/s41467-024-54809-7 (PMC11785797; doi:10.1038/s41467-024-54809-7)
Supplement: Supplementary file 1 — Supplementary Information [file 41467_2024_54809_MOESM1_ESM.pdf]

SUPPLEMENTAL INFORMATION

NSm is a critical determinant for bunyavirus transmission between vertebrate and mosquito hosts

a

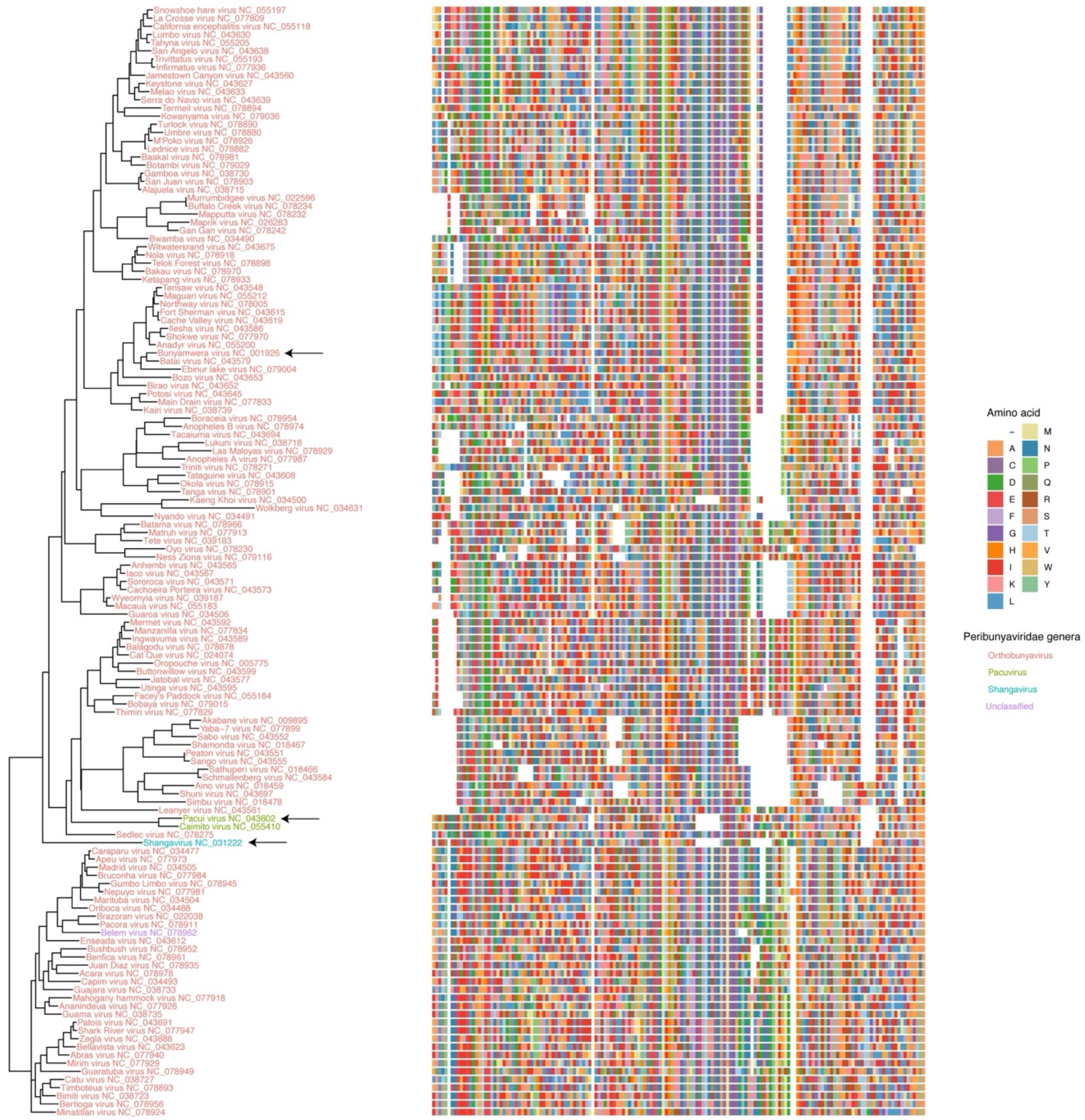

b

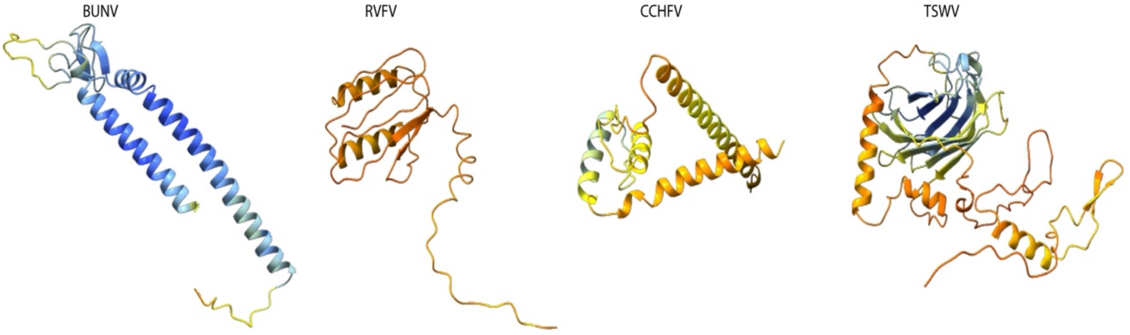

Figure S1. Phylogeny of NSm in *Peribunyaviridae* and 3D structure prediction of NSm proteins. a) Maximum likelihood phylogeny of the NSm protein sequences that have homology to the BUNV NSm mature protein with the corresponding amino acid alignment represented to the right. Distance mean: 0.8539, minimum distance: 0.1502, maximum distance: 0.9396. b) NSm protein structure prediction of BUNV, RVFV, CCHFV and TSWV based on colabfold (PMID: 35637307) with the colour representing the local confidence in the structure (blue – very high, light blue – high, yellow – low, orange – very low).

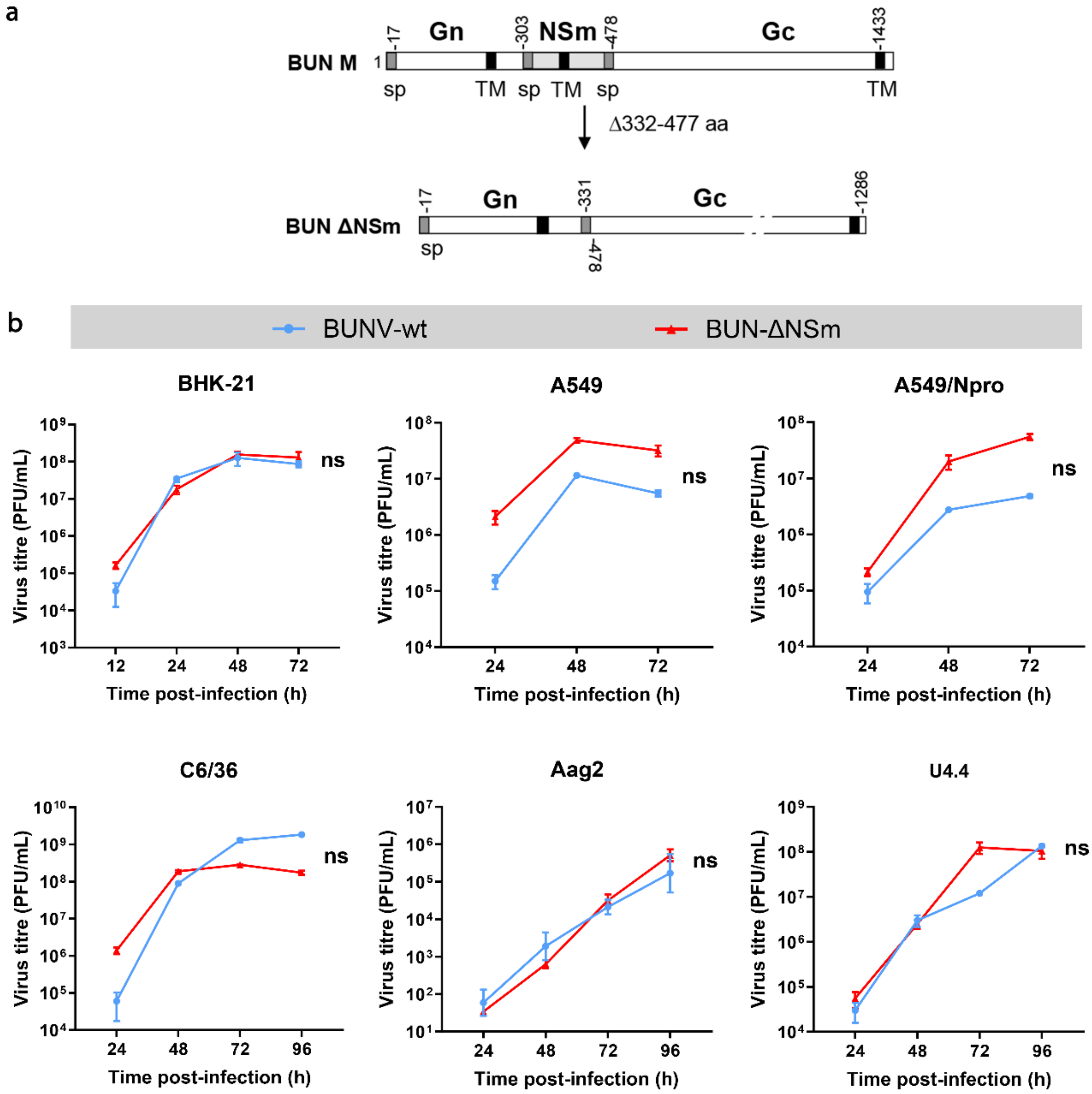

Figure S2. Replication of the recombinant BUNV NSm deletion virus in different cell types. (a) Schematic showing M segment of BUNV wild type (BUNV-wt) and BUNV NSm deletion (BUN-ΔNSm) viruses. In BUN-ΔNSm, the coding region for mature NSm and Gc signal peptide sequence (residues 332 to 477) were deleted giving rise to an M segment in which the NSm signal peptide (303 to 331) serves as signal peptide for Gc (starting at 478). (b) Growth curves of BUNV-wt and BUN-ΔNSm on mammalian cells (BHK-21, A549 and A549/Npro) and mosquito cells (*Ae. albopictus* larval C6/36 and U4.4 cell lines and *Ae. aegypti* embryonic Aag2 cell line). Cells were infected with BUNV (MOI 0.01), and culture supernatants were harvested at different time points post-infection as indicated. Viral titres were determined by plaque assay on BSR-T7/5 cells. Curves represent one

experiment performed in triplicate. Error bars represent standard errors of the means (SEM). Statistical testing by Mann-Whitney Test. ns = not significant.

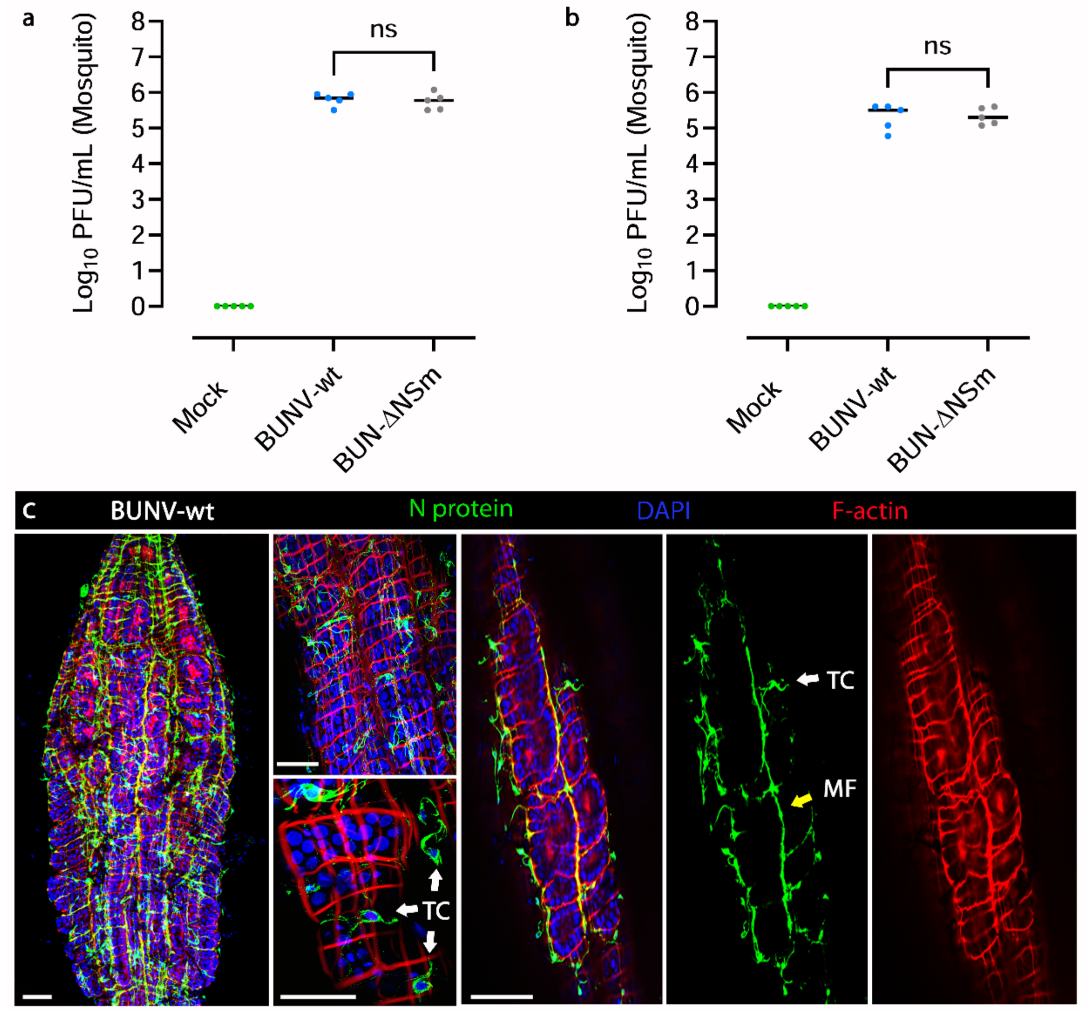

Figure S3. BUNV-wt and BUN-ΔNSm infection following blood feeding and intra-thoracic injection of *Ae. aegypti*. (a) Adult females were fed with an artificial blood meal containing 4 x 10^8 PFU/mL of BUNV-wt or BUN-ΔNSm and several whole mosquitoes were sampled minutes after blood feeding to ensure that the mosquitoes were fed with an equivalent number of viral particles from each viral strain. (b) Adult females were injected intrathoracically with 5 x 10^4 PFU/mosquito of BUNV-wt or BUN-ΔNSm and several whole mosquitoes were analysed minutes after injection to ensure that an equivalent number of viral particles of each viral strain were delivered in each mosquito. (a,b) Titres are displayed as Log_{10} PFU/mL with individual samples displayed (n = 5 per condition). Lines indicate median values and statistical significance shown on the graph was obtained using a two-tailed Mann-Whitney test between BUNV-wt and BUN-ΔNSm. ns, not significant. Source data are provided as a Source Data file. (c) Midguts were dissected at 3 dpi and stained with anti-N recognizing the viral nucleocapsid N protein

(green), Phalloidin Texas Red to visualize F-actin filaments (red) and DAPI to visualize nuclei (blue). Maximal intensity z-projection of a whole midgut and high-magnification images showing strong N expression in tracheal cells (TC, white) and muscle fibres (MF, yellow arrow). Scale bars are 150 μm.

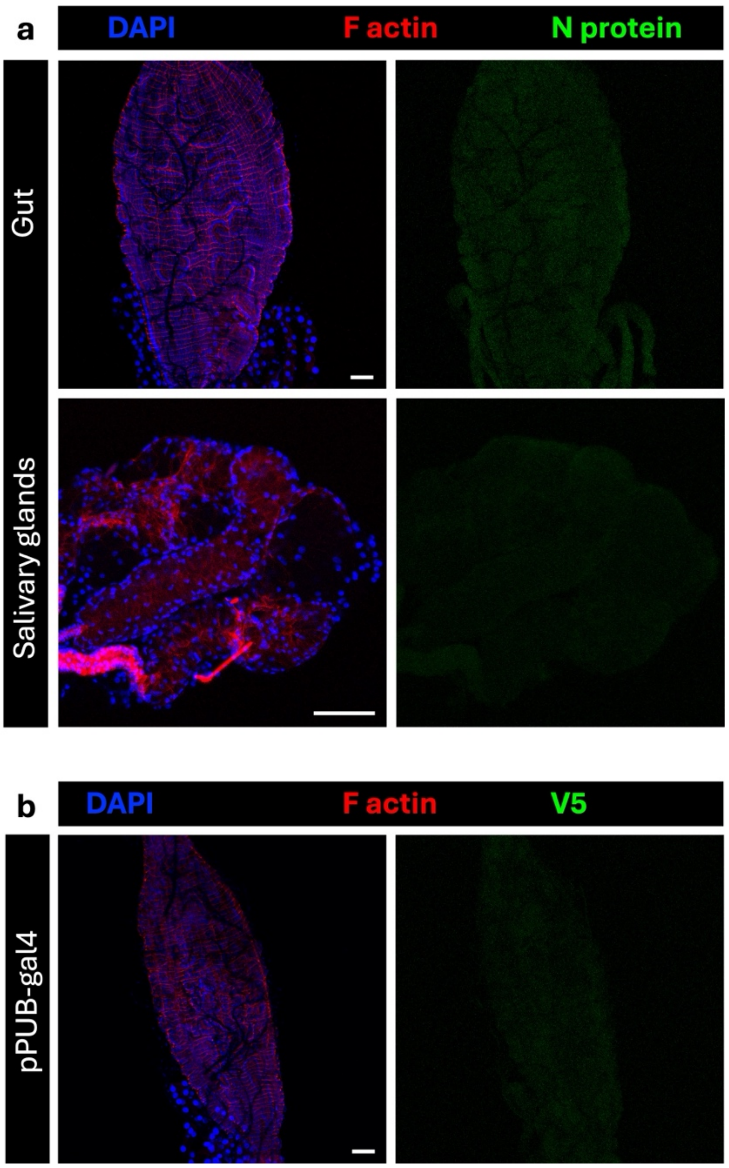

Figure S4. Anti-N and anti-V5 control immunostainings. (a) Midguts and salivary glands from non-infected females were dissected at 9 days post blood feeding and stained with anti-N recognizing the viral nucleocapsid N protein (green), Phalloidin Texas Red to visualize F-actin filaments (red) and DAPI to visualize nuclei (blue). (b) Mosquitoes were transfected with the plasmid pPUb-gal4. Guts were dissected at 6 days post injection and stained with anti-V5 (green), Phalloidin Texas Red (red) and DAPI (blue). Images are maximum intensity projection of Z-stacks and scale bars are 100µM.

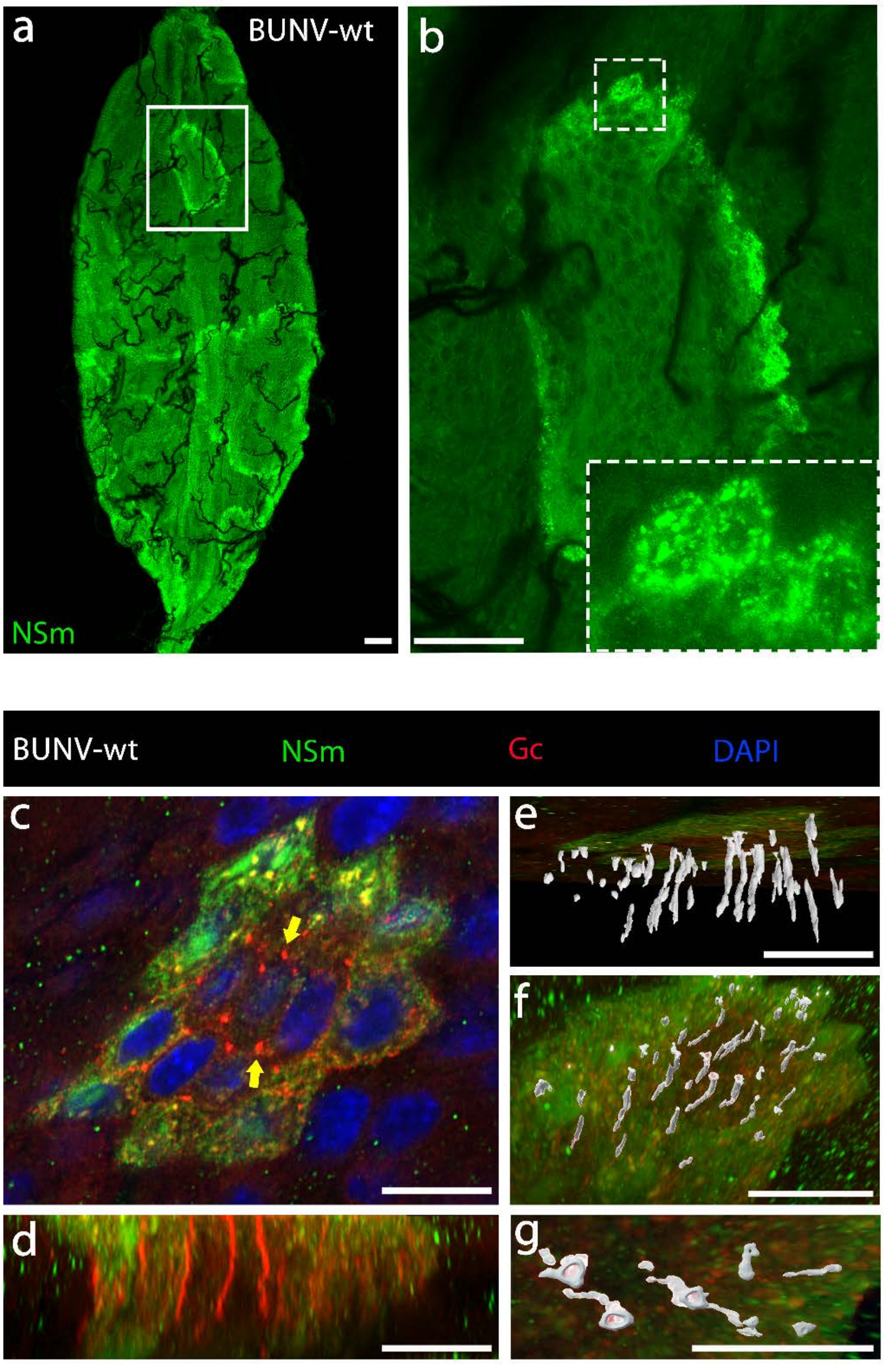

Figure S5. NSm expression in the midgut of BUNV infected *Ae. aegypti* mosquitoes. Mosquitoes were fed with blood containing 7 x 10^7 PFU/mL of BUNV-wt and midguts dissected at 3 dpbm. (a) Merged z-stacks image of a midgut stained with a BUNV-specific NSm antibody. (b) High-magnification image of the boxed area in (a) shows NSm immunoreactivity at the periphery of large infection foci. Panel contained an additional dashed boxed area to illustrate the NSm punctate staining of secretory vesicles. (c) Midgut infection focus co-stained with anti-NSm (green) and anti-Gc (red) and with DAPI to visualize nuclei (blue). Partial co-localisation was seen between NSm and Gc in

infected cells at the periphery of the cluster of cells. Yellow arrows show intense Gc expression localized to the tricellular corners of the hexagonally shaped epithelial cells. (d) Side view showing Gc immunoreactive signal concentrated between the cells. (e-g) Imaris surface rendering of Gc signal. (e) Bottom view, and (f) top view. (g) High-magnification 3-D image of the stalagmite-like Gc staining showing epithelium disruption at tricellular corner site. All scale bars are 100 μm.

Table S1. Primer sequences used in this study.

**Primer name**	**Primer sequence 5′->3′**	**Reference**
**pPUb-NSm-V5**		
pPUb-V5_F	GGTCGCAGCAGGCAAGCCCATCCCTAACCCACTG	This study
pPUb-V5_R	TGATCAATGACATGGTTGAAATCTCTGTTGAGCAG AAAAAGAAAC	This study
NSm-V5_F	TTCAACCATGTCATTGATCATATCCATCTTACTCTC	This study
NSm-V5_R	GGCTTGCCTGCTGCGACCATTATAATTGTATTC	This study
**pPUb-NSm**		
pPUb_F	GGTCGCAGCACTGATTCTAGAGTCGGGGCGGCCGG	This study
pPUb_R	TGATCAATGACATGGTTGAAATCTCTGTTGAGCAG AAAAAGAAACG	This study
NSm_F	TTCAACCATGTCATTGATCATATCCATCTTACTCTC	This study
NSm_R	CTAGAATCATGCTGCGACCATTATAATTGTATTC	This study
**RT-qPCR**		
BUNV-S_F	CAGTTGTCTCTAGCTTAGGTTGG	[1]
BUNV-S_R	ACATGTTGATTCCGAATTTAGC	This study
S7_F	CCAGGCTATCCTGGAGTTG	[2]
S7_R	GACGTGCTTGCCGGAGAAC	[2]

1. Feng, J., et al., *Interferon-Stimulated Gene (ISG)-Expression Screening Reveals the Specific Antibunyaviral Activity of ISG20.* J Virol, 2018. **92**(13).
2. McFarlane, M., et al., *Characterization of Aedes aegypti Innate-Immune Pathways that Limit Chikungunya Virus Replication.* PLOS Neglected Tropical Diseases, 2014. **8**(7): p. e2994.
