# Supplementary material for: NSm is a critical determinant for bunyavirus transmission between vertebrate and mosquito hosts
Source: Nat Commun. 2025 Jan 31;16:1214. doi: 10.1038/s41467-024-54809-7 (PMC11785797; doi:10.1038/s41467-024-54809-7)
Supplement: Supplementary file 2 — Reporting Summary [file 41467_2024_54809_MOESM2_ESM.pdf]

nature portfolio

Corresponding author(s): Emilie Pondeville and Xiaohong Shi

Last updated by author(s): 19/09/2024

Reporting Summary

Nature Portfolio wishes to improve the reproducibility of the work that we publish. This form provides structure for consistency and transparency in reporting. For further information on Nature Portfolio policies, see our Editorial Policies and the Editorial Policy Checklist.

Statistics

For all statistical analyses, confirm that the following items are present in the figure legend, table legend, main text, or Methods section.

n/a	Confirmed	
☐	☒	The exact sample size (n) for each experimental group/condition, given as a discrete number and unit of measurement
☐	☒	A statement on whether measurements were taken from distinct samples or whether the same sample was measured repeatedly
☐	☒	The statistical test(s) used AND whether they are one- or two-sided<br>*Only common tests should be described solely by name; describe more complex techniques in the Methods section.*
☒	☐	A description of all covariates tested
☐	☒	A description of any assumptions or corrections, such as tests of normality and adjustment for multiple comparisons
☐	☒	A full description of the statistical parameters including central tendency (e.g. means) or other basic estimates (e.g. regression coefficient) AND variation (e.g. standard deviation) or associated estimates of uncertainty (e.g. confidence intervals)
☐	☒	For null hypothesis testing, the test statistic (e.g. F, t, r) with confidence intervals, effect sizes, degrees of freedom and P value noted<br>*Give P values as exact values whenever suitable.*
☒	☐	For Bayesian analysis, information on the choice of priors and Markov chain Monte Carlo settings
☒	☐	For hierarchical and complex designs, identification of the appropriate level for tests and full reporting of outcomes
☒	☐	Estimates of effect sizes (e.g. Cohen's d, Pearson's r), indicating how they were calculated

Our web collection on statistics for biologists contains articles on many of the points above.

Software and code

Policy information about availability of computer code

Data collection	Images were acquired on a Zeiss LSM 880 inverted confocal microscope with Airyscan using the Zen or Imaris image analysis software packages and processed with Adobe Photoshop.<br>qPCR was performed with the QS3 Real Time PCR System (Applied Biosystems).<br>The mature peptide sequence of BUNV NSm was used to blast v2.11.0 using tblastn (PMID: 9254694) against all Bunyavirales reference genomes downloaded from Genbank on the 22nd of July 2024. The protein alignment of NSm was produced for all species with a match using MAFFT v7.475 (PMID: 23329690). Non-homologous gap regions of the alignment were removed using trimal v1.5 with option -gappyout (PMID: 19505945). The maximum likelihood tree was reconstructed in IQTREE version 2.1.3 (PMID: 32011700) using the best model based on ModelFinder and ultrafast bootstrap with 1000 replicates. The phylogeny and the alignment were visualized in R v4.4.0 using the packages APE (PMID: 30016406) and ggtree (PMID: 32162851) and pairwise distance calculated with the seqinr package [59]. Structures were predicted for the NSm sequences of BUNV, RVF, CCHFV and TSWV using the ColabFold (v1.5.5) implementation of AlphaFold2 (v2.3).
Data analysis	Statistical analysis was performed using a statistical software package (GraphPad Prism 9 and 10).

For manuscripts utilizing custom algorithms or software that are central to the research but not yet described in published literature, software must be made available to editors and reviewers. We strongly encourage code deposition in a community repository (e.g. GitHub). See the Nature Portfolio guidelines for submitting code & software for further information.

Data

Policy information about availability of data

All manuscripts must include a data availability statement. This statement should provide the following information, where applicable:

- Accession codes, unique identifiers, or web links for publicly available datasets
- A description of any restrictions on data availability
- For clinical datasets or third party data, please ensure that the statement adheres to our policy

The datasets generated and analysed during the current study are available in the University of Glasgow repository (http://dx.doi.org/10.5525/gla.researchdata.1516).

Research involving human participants, their data, or biological material

Policy information about studies with human participants or human data. See also policy information about sex, gender (identity/presentation), and sexual orientation and race, ethnicity and racism.

Reporting on sex and gender	NA
Reporting on race, ethnicity, or other socially relevant groupings	NA
Population characteristics	NA
Recruitment	NA
Ethics oversight	NA

Note that full information on the approval of the study protocol must also be provided in the manuscript.

Field-specific reporting

Please select the one below that is the best fit for your research. If you are not sure, read the appropriate sections before making your selection.

☒ Life sciences ☐ Behavioural & social sciences ☐ Ecological, evolutionary & environmental sciences

For a reference copy of the document with all sections, see nature.com/documents/nr-reporting-summary-flat.pdf

Life sciences study design

All studies must disclose on these points even when the disclosure is negative.

Sample size	No sample size was calculated. Sample size was chosen to fit with classical sample size used in the field.
Data exclusions	No data were excluded from the analyses.
Replication	All titration experiments were conducted on n>15 individual mosquitoes per group. All imagery was performed on at least 10 tissues aper group. qPCR were performed on 5 pools of 4 tissues per group. Experiments were repeated using at least independent batches of mosquitoes. All attempts at replication were successful.
Randomization	Mosquitoes were allocated randomly from different cages / blood feeding boxes to experimental /time point groups. Mosquitoes and samples infected with different viruses were marked on boxes / tubes along the experiment.
Blinding	Blinding was not possible due to one person doing a whole experiment.

Reporting for specific materials, systems and methods

We require information from authors about some types of materials, experimental systems and methods used in many studies. Here, indicate whether each material, system or method listed is relevant to your study. If you are not sure if a list item applies to your research, read the appropriate section before selecting a response.

Materials & experimental systems

n/a	Involved in the study	
☐	☒	Antibodies
☐	☒	Eukaryotic cell lines
☒	☐	Palaeontology and archaeology
☐	☒	Animals and other organisms
☒	☐	Clinical data
☒	☐	Dual use research of concern
☒	☐	Plants

Methods

n/a	Involved in the study	
☒	☐	ChIP-seq
☒	☐	Flow cytometry
☒	☐	MRI-based neuroimaging

Antibodies

Antibodies used	Rabbit polyclonal N antibody. Bleed 593 obtained from Richard Elliott. Watret, G.E., C.R. Pringle, and R.M. Elliott, Synthesis of bunyavirus-specific proteins in a continuous cell line (XTC-2) derived from Xenopus laevis. J Gen Virol, 1985. 66 (Pt 3): p. 473-82.<br><br>Mouse monoclonal V5 tag antibody. Abcam [SV5-Pk1] ab27671.<br><br>Rabbit polyclonal anti-NSm. Obtained from Richard Elliott. Nakitare, G.W. and R.M. Elliott, Expression of the Bunyamwera Virus M Genome Segment and Intracellular Localization of NSm. Virology, 1993. 195(2): p. 511-520.<br><br>Mouse monoclonal anti-Gc. Anti-G1 Mab810 obtained from Richard Elliott. Lappin, D.F., et al., Localization of Bunyamwera bunyavirus G1 glycoprotein to the Golgi requires association with G2 but not with NSm. J Gen Virol, 1994. 75 (Pt 12): p. 3441-51.
Validation	Rabbit polyclonal N antibody. Watret, G.E., C.R. Pringle, and R.M. Elliott, Synthesis of bunyavirus-specific proteins in a continuous cell line (XTC-2) derived from Xenopus laevis. J Gen Virol, 1985. 66 (Pt 3): p. 473-82.<br><br>Mouse monoclonal V5 tag antibody [SV5-Pk1] ab27671. https://www.abcam.com/en-us/products/primary-antibodies/v5-tag-antibody-sv5-pk1-ab27671#tab=support<br><br>Rabbit polyclonal anti-NSm. Nakitare, G.W. and R.M. Elliott, Expression of the Bunyamwera Virus M Genome Segment and Intracellular Localization of NSm. Virology, 1993. 195(2): p. 511-520.<br><br>Mouse monoclonal anti-Gc. Lappin, D.F., et al., Localization of Bunyamwera bunyavirus G1 glycoprotein to the Golgi requires association with G2 but not with NSm. J Gen Virol, 1994. 75 (Pt 12): p. 3441-51.

Eukaryotic cell lines

Policy information about cell lines and Sex and Gender in Research

Cell line source(s)	Mosquito Ae. albopictus C6/36 and U4.4 cell lines (derived from larvae, obtained from Prof. A. Kohl, LSTM, UK) - unknown sex.<br>Walker, T., et al., Mosquito cell lines: history, isolation, availability and application to assess the threat of arboviral transmission in the United Kingdom. Parasit Vectors, 2014. 7: p. 382.<br>Condreay, L.D. and D.T. Brown, Exclusion of superinfecting homologous virus by Sindbis virus-infected Aedes albopictus (mosquito) cells. J Virol, 1986. 58(1): p. 81-6.<br><br>Mosquito Ae. aegypti Aag2 cell line (derived from embryonic tissue, obtained from Prof. P. Eggleston, Keele University, UK). Unkown sex.<br>Walker, T., et al., Mosquito cell lines: history, isolation, availability and application to assess the threat of arboviral transmission in the United Kingdom. Parasit Vectors, 2014. 7: p. 382.<br><br>Hamster BHK-21 cells (obtained from Prof. R. M. Elliott, MRC-University of Glasgow Centre for Virus Research, UK) - Male<br><br>BSR-T7/5 cells (obtained from Prof. R. M. Elliott, MRC-University of Glasgow Centre for Virus Research, UK) - Male<br>Buchholz, U.J., S. Finke, and K.K. Conzelmann, Generation of bovine respiratory syncytial virus (BRSV) from cDNA: BRSV NS2 is not essential for virus replication in tissue culture, and the human RSV leader region acts as a functional BRSV genome promoter. J Virol, 1999. 73(1): p. 251-9.<br>A549 and A549/NPro cell lines (obtained from Prof. R. E. Randall, University of St. Andrews, UK) - Male
Authentication	None of the cell lines were authenticated.
Mycoplasma contamination	Cells were not tested for mycoplasma.
Commonly misidentified lines<br>(See ICLAC register)	NA

Animals and other research organisms

Policy information about studies involving animals; ARRIVE guidelines recommended for reporting animal research, and Sex and Gender in Research

Laboratory animals	Mosquitoes: Aedes aegypti, Paea laboratory strain
Wild animals	NA
Reporting on sex	Findings only applied to female mosquitoes as they blood feed while males do not.
Field-collected samples	NA
Ethics oversight	No ethical approval required as animals used in the study are insects.

Note that full information on the approval of the study protocol must also be provided in the manuscript.

Plants

Seed stocks	NA
Novel plant genotypes	NA
Authentication	NA
